# The antipsychotic agent sulpiride microinjected into the ventral pallidum restores positive symptom-like habituation disturbance in MAM-E17 schizophrenia model rats

**DOI:** 10.1038/s41598-024-63059-y

**Published:** 2024-05-29

**Authors:** László Péczely, Daniella Dusa, László Lénárd, Tamás Ollmann, Erika Kertes, Rita Gálosi, Beáta Berta, Ádám Szabó, Kristóf László, Olga Zagoracz, Zoltán Karádi, Veronika Kállai

**Affiliations:** 1 Learning in Biological and Artificial Systems Research Group, Institute of Physiology, Medical School, University of Pécs, Pécs, Hungary; 2 Neuropeptides, Cognition, Animal Models of Neuropsychiatric Disorders Research Group, Institute of Physiology, Medical School, University of Pécs, Pécs, Hungary; 3 Reinforcement Learning Research Group, Institute of Physiology, Medical School, University of Pécs, Pécs, Hungary; 4 Present Address: Institute of Physiology, Medical School, University of Pécs, Szigeti Str. 12, P.O. Box: 99, 7602 Pécs, Hungary; 5 Molecular Neuroendocrinology and Neurophysiology Research Group, Szentágothai Research Centre, University of Pécs, Pécs, Hungary; 6 Centre for Neuroscience, University of Pécs, Pécs, Hungary

**Keywords:** Schizophrenia, D2-like DA receptors, Ventral pallidum, MAM-E17 rats, Sulpiride, Habituation disturbance, Reward, Reward, Neuroscience, Diseases of the nervous system, Psychosis, Schizophrenia

## Abstract

Dysfunction of subcortical D2-like dopamine receptors (D_2_Rs) can lead to positive symptoms of schizophrenia, and their analog, the increased locomotor activity in schizophrenia model MAM-E17 rats. The ventral pallidum (VP) is a limbic structure containing D_2_Rs. The D_2_R antagonist sulpiride is a widespread antipsychotic drug, which can alleviate positive symptoms in human patients. However, it is still not known how sulpiride can influence positive symptoms via VP D_2_Rs. We hypothesize that the microinjection of sulpiride into the VP can normalize hyperactivity in MAM-E17 rats. In addition, recently, we showed that the microinjection of sulpirid into the VP induces place preference in neurotypical rats. Thus, we aimed to test whether intra-VP sulpiride can also have a rewarding effect in MAM-E17 rats. Therefore, open field-based conditioned place preference (CPP) test was applied in neurotypical (SAL-E17) and MAM-E17 schizophrenia model rats to test locomotor activity and the potential locomotor-reducing and rewarding effects of sulpiride. Sulpiride was microinjected bilaterally in three different doses into the VP, and the controls received only vehicle. The results of the present study demonstrated that the increased locomotor activity of the MAM-E17 rats was caused by habituation disturbance. Accordingly, larger doses of sulpiride in the VP reduce the positive symptom-analog habituation disturbance of the MAM-E17 animals. Furthermore, we showed that the largest dose of sulpiride administered into the VP induced CPP in the SAL-E17 animals but not in the MAM-E17 animals. These findings revealed that VP D_2_Rs play an important role in the formation of positive symptom-like habituation disturbances in MAM-E17 rats.

## Introduction

Schizophrenia is a widespread, devastating psychiatric disease^1^. The MAM-E17 schizophrenia rat model is one of the most widely accepted animal models of schizophrenia and recapitulates some of the positive, negative, and cognitive symptoms of the disease^2–4^. It was shown that there is an enhanced response to psychomotor stimulants, such as the indirect dopamine (DA) agonist amphetamine, in schizophrenia^5^ and in rodent models^3,6^. DAergic hyperresponsivity is postulated to be responsible for the positive symptoms of schizophrenia^7^. Hippocampal hyperactivity correlates with the presence of psychosis in individuals with schizophrenia^8^, while in MAM-E17 rats, hippocampal overactivity leads to hyperresponsivity of the DAergic system and results in increased locomotor activity^6^, which can be paralleled with the positive symptoms of the disease^9,10^. It was shown that hyperactivity of the glutamatergic output neurons of the hippocampus leads to increased DAergic population activity in the ventral tegmental area (VTA)^6^ via the nucleus accumbens (NAC)-ventral pallidal (VP) axis^11^.

D_2_ dopamine receptors (D_2_Rs) play an important role in triggering primarily the positive symptoms of schizophrenia^7,12–14^ but also cognitive disturbances^15^. Most current therapeutic drugs for schizophrenia bind to D_2_Rs and reduce aberrant dopamine (DA) transmission^16^. Chronic administration of D_2_R antagonist antipsychotic drugs effectively reduces the number of spontaneously active DAergic neurons via the “depolarization block” mechanism^17–20^. MAM-E17 rats display an increase in DA neuron activity and an aberrant locomotor response in response to the D2R agonist quinpirole; furthermore, a significant increase in D_3_R (D_3_R, which belongs to the D_2_R group) mRNA expression can be observed in the NAC, which suggests that these receptors are likely responsible for the sensitized locomotor-activating response to quinpirole^21^. In addition to the enhanced VTA DAergic population activity and the increased number of D_2_Rs, an elevation in D_2_ High receptors, which possess high-affinity state for DA, can also be observed in schizophrenia patients^22^. D_2_R-expressing NAC neurons project to the VP, and their stimulation facilitates VTA DAergic activity^23^. The VP, an element of the hippocampus-NAC-VP-VTA axis, is the main regulator of the population activity of DAergic neurons in the VTA^24^. Nevertheless, less is known about the role of VP D_2_Rs in the pathomechanism of schizophrenia.

D_2_Rs can be found pre- and postsynaptically in the VP^25–27^. We recently showed that VP D_2_Rs exert negative regulatory feedback on the VTA DAergic population and burst activity^28^. The D_2_R antagonist sulpiride dose-dependently impaired memory consolidation in spatial learning in rats^29^. Furthermore, in the VP, the D_2_R agonist quinpirole dose-dependently induces place aversion^28^, while the intra-VP sulpiride induces place preference and decreases locomotor activity in experimental animals^29^.

Based on these experimental findings, the question arises of how D_2_R antagonists microinjected into the VP affect the positive symptom-analogue increased locomotor activity in MAM-E17 schizophrenia model rats. According to our hypothesis, D_2_R antagonist antipsychotic medications, such as sulpiride, could exert their effects at least partly via VP D_2_Rs, presumably reducing hyperactivity in MAM-E17 rats. On the other hand, since the administration of sulpiride to the VP induces place preference in rats^29^, we can suppose that it has similar effects in MAM-E17 rats.

Therefore, to test our hypothesis and investigate the potential locomotor and rewarding effects of sulpiride in the VP of neurotypical (SAL-E17) and MAM-E17 animals, open field-based conditioned place preference (CPP) test was performed.

## Methods

### Animals

The experimental rats were bred in our laboratory. The estrous cycle of female Wistar rats was monitored (Charles River, Hungary), and on the evening of proestrus, the rats were paired. On the 17th day of pregnancy, Wistar dams were treated with methylazoxymethanol acetate (MRIGlobal Chemical Carcinogen Repository, Kansas City, Missouri; 25 mg/kg dissolved in saline) or 0.9% physiological saline solution intraperitoneally (i.p.), similar to the procedure of Grace and Lodge^6,30,31^. Litters were weaned 4 weeks after birth. The male offspring of the i.p. MAM-injected dams were used in the experiments as the MAM-E17 schizophrenia model animals, while the male offspring of the i.p. saline-injected dams were the SAL-E17 rats. Female offspring were used in another experiment.

All animal experiments were conducted, and all animals were cared for according to federal and local ethical guidelines. The protocols were approved by the National Scientific Ethical Committee on Animal Experimentation of Hungary (BA02/2000-8/2012, BA02/2000-65/2017 and BA02/2000-64/2017, Pécs University, Medical School; Hungarian Government Decree, 40/2013). (II. 14.); NIH Guidelines, 1997; European Community Council Directive 86/609/EEC 1986, 2006; European Directive 2010/63/EU of the European Parliament). The present study is reported in accordance with the ARRIVE guidelines.

Housing rooms were maintained at a standard temperature (21 ± 2 °C) and on a light–dark cycle (12:12 h light–dark cycle with lights on at 7:00 a.m.). Standard laboratory food pellets (CRLT/N standard rodent food pellet, Charles River Kft, Budapest, Hungary) and tap water were available ad libitum. Every effort was made to minimize the number and suffering of the animals used.

Behavioral tests were performed during the daylight cycle between 08:00 and 18:00 h. The experiments were carried out only on male offspring (SAL-E17 rats: n = 37; MAM-E17 rats: n = 41; respectively).

### Stereotaxic surgery

Stereotaxic surgery was performed under general anesthesia by intraperitoneal injection of ketamine and diazepam (Calypsol: 80 mg/kg bw. and Seduxen, 20 mg/kg bw., respectively; Richter Gedeon Ltd., Hungary).

For drug administration, guide cannulae (made of 22-gauge stainless steel tubes) were implanted bilaterally 0.5 mm above the VP. The coordinates were determined according to the rat brain’s stereotaxic atlas^32^. (Coordinates relative to bregma: AP: − 0.3 mm; ML: ± 2.2 mm; V: − 7.1 mm from dura). Cannulae were fixed with self-polymerizing dental acrylic (Duracryl), which was anchored by 3 stainless steel screws screwed into the skull. The guide cannulae were secured with stainless steel obturators made of 27-gauge stainless steel wire. After surgery, the animals were given one week to recover before the experiments began.

### Drug administration and microinjection procedure

The D2R antagonist sulpiride (Sigma‒Aldrich Co.: (S)-(-)-Sulpiride, S7771) was bilaterally microinjected in three different doses: 0.1 μg, 1.0 μg, and 4.0 μg per side in a 0.4 μl volume (0.73 mM, 7.32 mM, and 29.29 mM, respectively). Sulpiride was dissolved in 0.1 N HCl, and after the addition of phosphate buffer, it was titrated with 0.1 N NaOH. The control animals received this solution bilaterally as a vehicle in an equal volume to that used for the sulpiride injections. Test solutions were stored at + 4 °C before application.

Based on the dose of drug injected, both SAL-E17 and MAM-E17 animals were divided into four groups (cohorts): SAL-E17 rats: (1) vehicle, n = 9 (SAL-E17 veh); (2) 0.1 μg sulpiride, n = 7 (SAL-E17 0.1 μg); (3) 1.0 μg sulpiride, n = 9 (SAL-E17 1.0 μg); (4) 4.0 μg sulpiride, n = 8 (SAL-E17 4.0 μg); and MAM-E17 rats: (1) vehicle, n = 10 (MAM veh); (2) 0.1 μg sulpiride, n = 9 (MAM 0.1 μg); (3) 1.0 μg sulpiride, n = 9 (MAM 1.0 μg); and (4) 4.0 μg sulpiride, n = 10 (MAM 4.0 μg); respectively (after histology). The experimental groups with treatments and number of animals are summarized in Table 1.

**Table 1:** Experimental cohorts and number of animals (n, after histology) in the CPP paradigm.

Treatment and dose		Number of animals (n)	Experimental cohort
Pseudoconditioning	Conditioning		
SAL-E17 cohorts			
Vehicle	Vehicle	9	SAL veh
Vehicle	0.1 μg sulpiride	7	SAL 0.1 μg
Vehicle	1.0 μg sulpiride	9	SAL 1.0 μg
Vehicle	4.0 μg sulpiride	8	SAL 4.0 μg
MAM-E17 cohorts			
Vehicle	Vehicle	10	MAM veh
Vehicle	0.1 μg sulpiride	9	MAM 0.1 μg
Vehicle	1.0 μg sulpiride	9	MAM 1.0 μg
Vehicle	4.0 μg sulpiride	10	MAM 4.0 μg

For further explanation, see the text.

During the microinjection procedure, the rats were gently held in the hand. Stainless-steel injection cannulae were inserted into the bilaterally implanted guide cannulae, which were attached to a 10 µl Hamilton microsyringe via a polyethylene tube. The injection cannulae protruded from the guide cannulae with 0.5 mm. The solutions were microinjected with a syringe pump (Cole Parmer, IITC, Life Sci. Instruments, California) in a volume of 0.4 μl for 60 s. After the injection, the injection cannulae were left in place for an additional 60 s to allow diffusion into the surrounding tissue and to prevent backflow along the insertion track. After drug (or vehicle) treatment, the rats were placed into the apparatus within 5 min.

### Locomotor activity and conditioned place preference measurements

The positive reinforcing effects of drugs and locomotor activity of the animals can be examined in the open field-based CPP paradigm^33,34^. The CPP apparatus consisted of an 85 cm diameter circular open field surrounded by a 40 cm high wall. The floor was divided by black lines into four quadrants of equal size. Visual cues in the environment helped the orientation of the animals inside the apparatus and allowed them to distinguish quadrants^33^. The behavior of the rats was recorded, and the stored data were analyzed by the Noldus EthoVision Basic video tracking system (EthoVision; Noldus Information Technology, The Netherlands). All trials were conducted in a sound-isolated, dimly illuminated experimental room.

**Figure 1:**
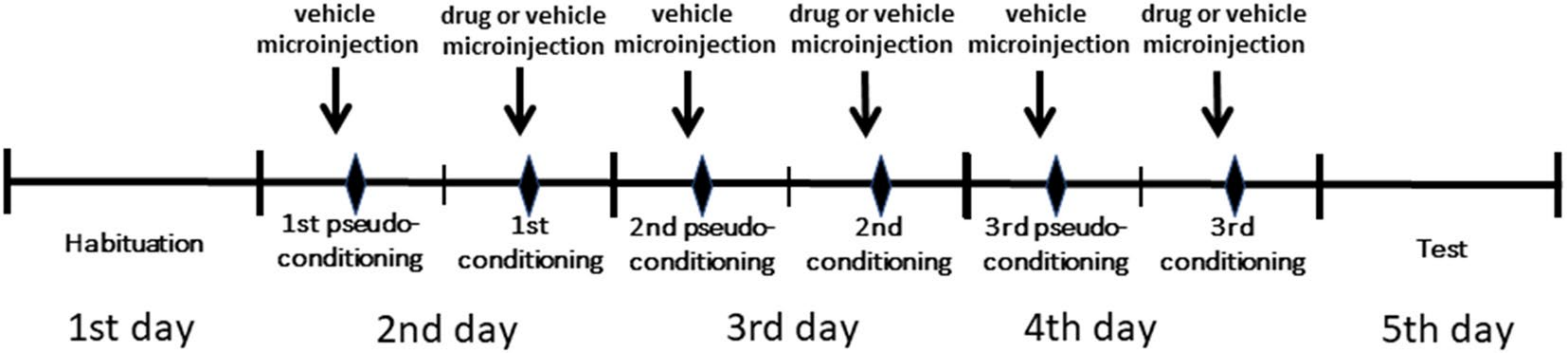
Schematic illustration showing the experimental procedure used for the CPP test. For further explanation, see the text.

The place preference procedure consisted of one habituation (1st day), three conditioning (2nd–4th days), and one test (5th day) day. The experimental procedure is illustrated in Fig. 1.

Pseudoconditioning trials were performed to make the animals able to discriminate that not the whole apparatus but only the conditioning quadrant is rewarding.

On the first experimental day (habituation trial), the animals were placed into the apparatus and allowed to freely move to all quadrants of the arena. On this day, the animals were not under drug treatment. Subsequently, during the 2nd–4th days (conditioning days), the rats were restricted to one quadrant by means of a Plexiglas barrier. For each animal, a neutral quadrant was assigned as the conditioning quadrant, i.e., one of the quadrants in which the animal had spent neither the shortest nor the longest time during habituation. The pseudoconditioning quadrant was the area opposite the conditioning quadrant. On the morning of the conditioning days, the rats were subjected to bilateral microinjection of vehicle and then immediately placed in the pseudoconditioning quadrant. In the afternoon, the rats were placed in the conditioning quadrant after receiving the appropriate drug microinjection. On the 5th day (test trial), the Plexiglas barrier was removed, and the animals were allowed to move freely through all parts of the apparatus without acute drug exposure. Each trial lasted for 900 s (15 min). The apparatus was cleaned after each session.

The time that the rats spent in each of the four quadrants was analyzed. If rats in the test trial spent more time in the drug-related quadrant, they were considered to have developed CPP. The locomotor activity of the rats was analyzed in the same apparatus under the same behavioral protocol by measuring the distance traveled by the rats during the habituation and test trials. In addition, we calculated the difference in the measured parameters (time spent in the conditioning quadrant and distance traveled) between the test and the habituation trials for each animal.

### Histology

Following the behavioral experiments, the rats were overdosed with urethane (20% urethane solution i.p. injection, in a dose of 1.4 g/kg bw.) and were transcardially perfused with 0.9% physiological saline solution followed by 10% formaldehyde solution. After one week of fixation, the brains were frozen, 40 μm sections of the VP were cut, and 60 μm coronal sections of the dorsal hippocampus were cut and then stained with cresyl violet. For microscopic analysis of the VP, the microinjection sites on the slides were reconstructed according to the rat brain stereotaxic atlas^32^. Only data from animals with correctly placed cannulae were analyzed. In the dorsal hippocampus containing slides, the pyramidal cell arrangement of the dorsal hippocampus was inspected. The brain slices were photographed with a Nikon Eclipse Ti2-E fluorescence confocal microscope.

### Statistical analysis

For statistical analysis, three-way and two-way mixed ANOVAs and two-way ANOVA were applied, and all were followed by Bonferroni post hoc test. In the case of three-way and two-way ANOVAs, intrauterine MAM/vehicle treatment and/or drug treatment (vehicle, 0.1 μg, 1.0 μg and 4.0 μg sulpiride) were the between-subject factors. To examine the possible correlations between the variables, the Pearson correlation test was used.

Statistical calculations were carried out with SPSS software. The significance level was defined as p < 0.05. The data are presented as the mean ± standard error of the mean (S.E.M.).

## Results

### Histology

**Figure 2:**
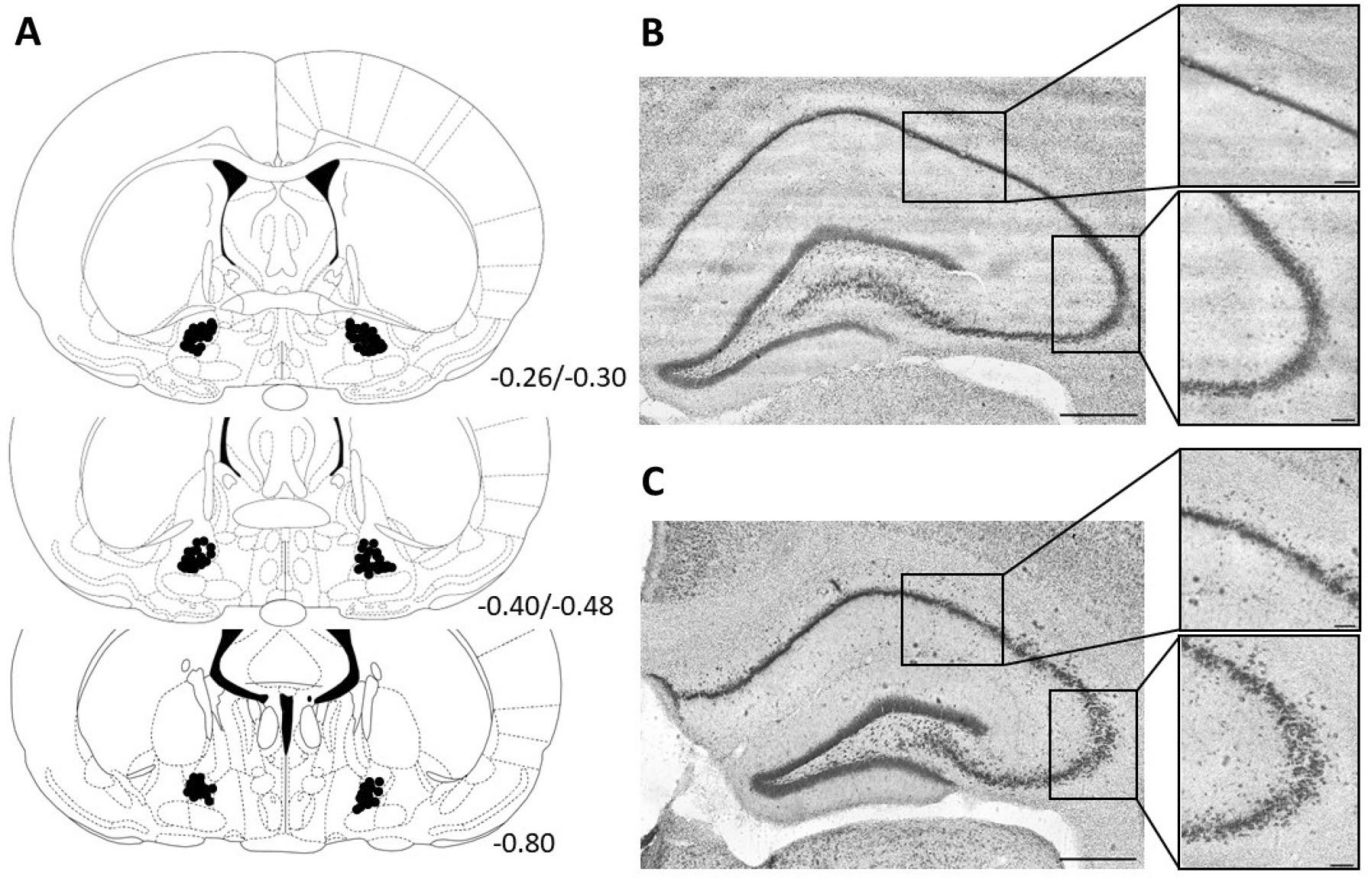
Results of the histological analysis. On the left side of the figure (**A**), a schematic representation of the correct placement of the bilateral guide cannulae in the VP is shown in a coronal section from the rat brain atlas of Paxinos and Watson. The numbers next to the sections indicate the anterior–posterior distance from the bregma in mm. On the right side, structural alterations in the dorsal hippocampus due to MAM-E17 treatment can be seen (**B**, **C**). Panel (**B**) shows the dorsal hippocampus at 10 × magnification and the CA1 and CA3 regions at 20 × magnification in a SAL-E17 rat. Panel (**C**) shows the same area in the case of a MAM-E17 rat. In the latter, disperse cell locations and heterotopias can be observed, which is typical in MAM-E17 schizophrenia model animals. Scale bars: 500 μm (complete picture) and 100 μm (zoomed picture).

Histological verification/analysis revealed that the guide cannulae were accurately and symmetrically positioned above the target area in 71 of the 78 animals. Cannula tracks and the location of the tips were determined based on the presence of debris from the destroyed elements and the degree of moderate glial proliferation. A schematic representation of the location of the cannula tips is shown in Fig. 2A. The remaining 7 rats were not included in the subsequent data analysis due to incorrect cannula placement. In 3 of those rats, the cannulae were in the horizontal diagonal band, 1 was caudal to the VP in the lateral hypothalamus (LH), 1 was too anterior in the caudate-putamen, and 2 were in the lateral preoptic area (data not shown).

To monitor the effectiveness of the MAM treatment, the dorsal hippocampal region of the MAM-E17 and SAL-E17 animals was also inspected via qualitative microscopic analysis (Fig. 2B,C). In the brains of all MAM-E17 rats, mild or definite disarray was observed in the pyramidal cell layer. In contrast to the regular compact structure observed in the SAL-E17 rats (Fig. 2B), disperse cell locations and heterotopia were found in the hippocampus of the MAM-E17 rats (Fig. 2C).

### Conditioned place preference

**Figure 3:**
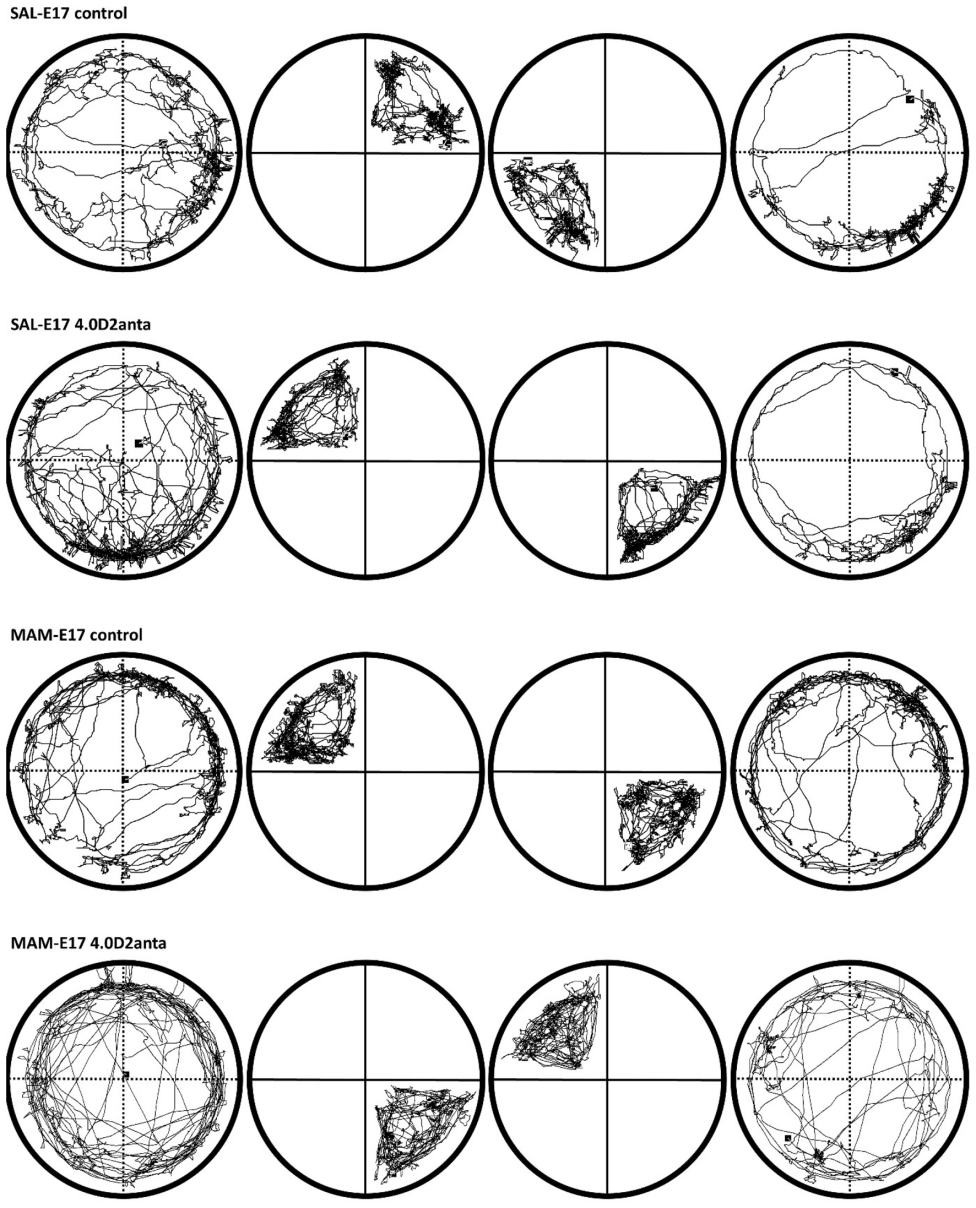
Representative example tracks of SAL-E17 and MAM-E17 control and 4.0 µg sulpiride-treated (4.0D2anta) animals in the CPP paradigm within the sketch of the experimental apparatus. The solid lines in the sketch of the experimental paradigms represent physical barriers (the plexiglass barriers in the CPP apparatus). Dashed lines indicate the boundaries of virtual spaces, i.e., the virtual quadrants in the CPP paradigm. In the first column, example tracks of the habituation trial are illustrated, while in the second and third columns, tracks of the first pseudoconditioning and conditioning trials can be seen, respectively. In the fourth column, example tracks of the test trial are displayed. In the test trial, the control and 4.0D2anta SAL-E17 rats moved less than they did in the habituation trial, but apparently, this effect was not observed for the MAM-E17 control rats. However, the 4.0 µg sulpiride treatment reduced effectively the locomotor activity of the MAM-E17 rats. The 4.0D2anta SAL-E17 rats spent significantly more time in the conditioning quadrant in the test trial than in the habituation trial.

**Figure 4:**
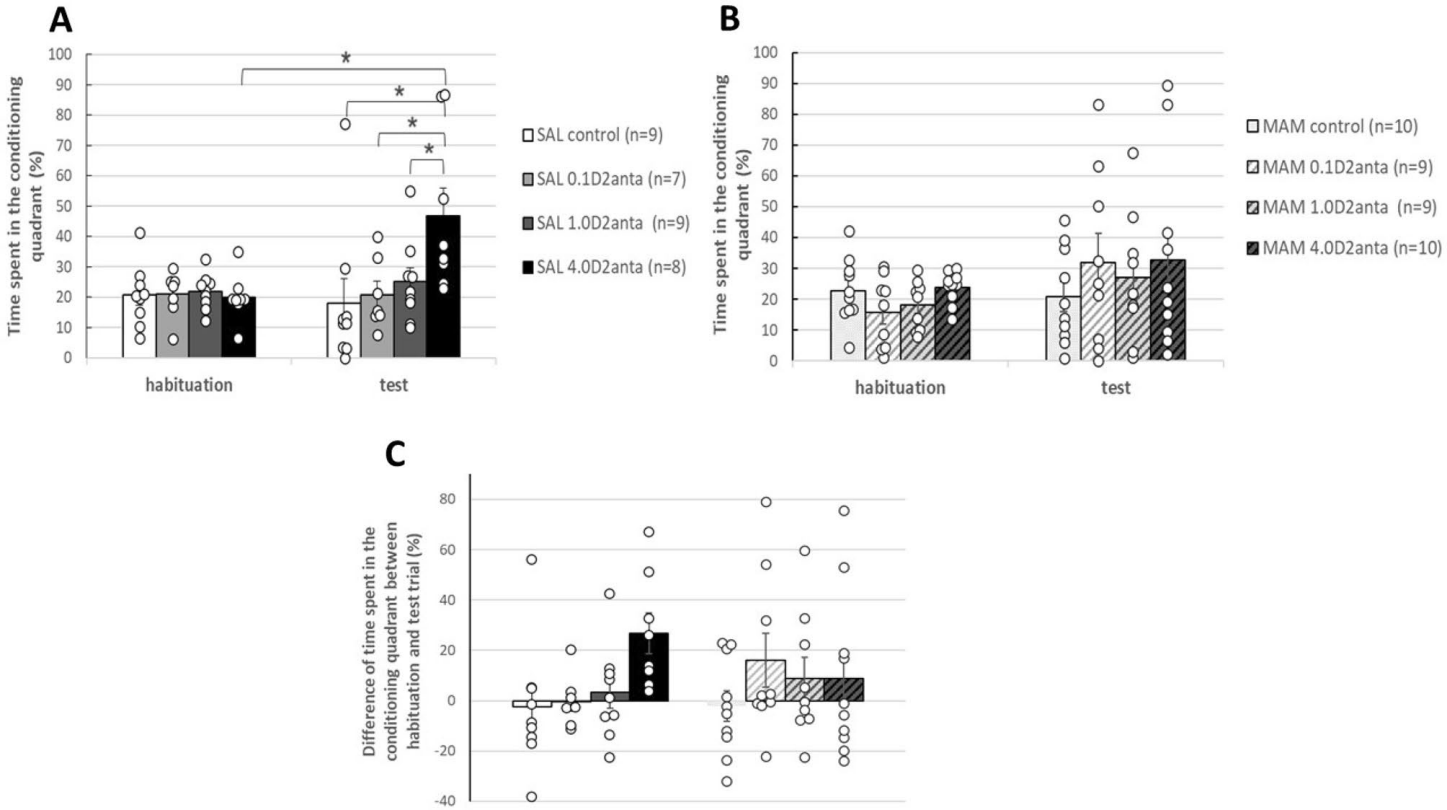
Effect of VP-sulpiride treatment on time spent (%) in the conditioning quadrant during the habituation and test trials in SAL-E17 rats (**A**) and MAM-E17 rats (**B**) in CPP paradigm. Panel C displays the difference in time spent in the conditioning quadrant between the test and habituation trials in both the SAL-E17 and MAM-E17 rats. The 4.0 µg dose of sulpiride induced place preference in the SAL-E17 group but not in the MAM-E17 group. The columns represent the average percentage (± S.E.M.) of time spent in the conditioning quadrant in panels (**A**) and (**B**), as well as the mean difference in time spent in the conditioning quadrant in percentage (± S.E.M.) between the habituation and test trials in panel (**C**). The results of the individual subjects (represented as circles) are added to the bar graphs. *p < 0.05 indicates significant differences.

The potential rewarding effect of sulpiride was examined in the CPP paradigm. Representative example tracks of the SAL-E17 and MAM-E17 control and 4.0 µg sulpiride-treated animals in the CPP paradigm are shown in Fig. 3, while the results for the SAL-E17 and MAM-E17 model rats in the CPP paradigm are shown in Fig. 4.

Three-way mixed ANOVA of the time spent in the conditioning quadrant revealed a nonsignificant intrauterine MAM/vehicle treatment effect [F(1,71) = 0.001; p = 0.978], a significant drug treatment effect [F(3,71) = 2.980; p = 0.037] and a significant effect of trial [F(1,71) = 6.922; p = 0.010]. Intrauterine MAM/vehicle treatment × drug treatment interaction (F(3,71) = 0.487; p = 0.692), intrauterine MAM/vehicle treatment × trial interaction (F(1,71) = 0.036; p = 0.850) and drug treatment × trial interaction (F(3,71) = 2.272; p = 0.088) were not significant. Intrauterine MAM/vehicle treatment x drug treatment x trial interaction (F(3,71) = 1.575; p = 0.203) was not significant. Post hoc test demonstrated that the rats of the 4.0D2anta treated groups spent more time in the conditioning quadrant than the other groups (control, 0.1D2anta and 1.0D2anta groups, p = 0.031, p = 0.038, p = 0.007, respectively). Furthermore, there was a significant difference between the habituation and the test trial (p = 0.010).

We have analyzed results of the SAL-E17 and MAM-E17 animals also separately, using two-way mixed ANOVA.

Two-way mixed ANOVA of the time spent in the conditioning quadrant by the SAL-E17 rats (Fig. 4A) revealed a significant drug treatment effect [F(3,33) = 2.905; p = 0.049], a nonsignificant effect of trial [F(1,33) = 4.063; p = 0.052], and a significant drug x trial interaction (F(3,33) = 3.930; p = 0.017). Post hoc tests revealed that the rats in the SAL-E17 4.0D2anta group spent more time in the conditioning quadrant in the test trial than did those in the other groups (control, 0.1D2anta and 1.0D2anta groups, p = 0.001, p = 0.005, p = 0.016, respectively). the SAL-E17 4.0D2anta group spent more time in the conditioning quadrant compared to the habituation trial (p = 0.001).

In the case of the MAM-E17 rats (Fig. 4B), however, two-way mixed ANOVA indicated a nonsignificant drug treatment effect [F(3,38) = 0.616; p = 0.609], a nonsignificant effect of trial [F(1,38) = 3.368; p = 0.074], and a nonsignificant drug x trial interaction (F(3,38) = 0.767; p = 0.520).

For each animal, the difference in the time spent in the conditioning quadrant between the test and habituation trials was also calculated and illustrated (Fig. 4C). Two-way ANOVA revealed a nonsignificant main effect of drug treatment [F(3, 63) = 2.016, p = 0.121] and a nonsignificant effect of intrauterine MAM/vehicle treatment [F(1, 63) = 0.032, p = 0.859]. Furthermore, a nonsignificant drug x intrauterine MAM/vehicle treatment interaction (F(3, 63) = 1.398, p = 0.252) was demonstrated.

### Locomotor activity

**Figure 5:**
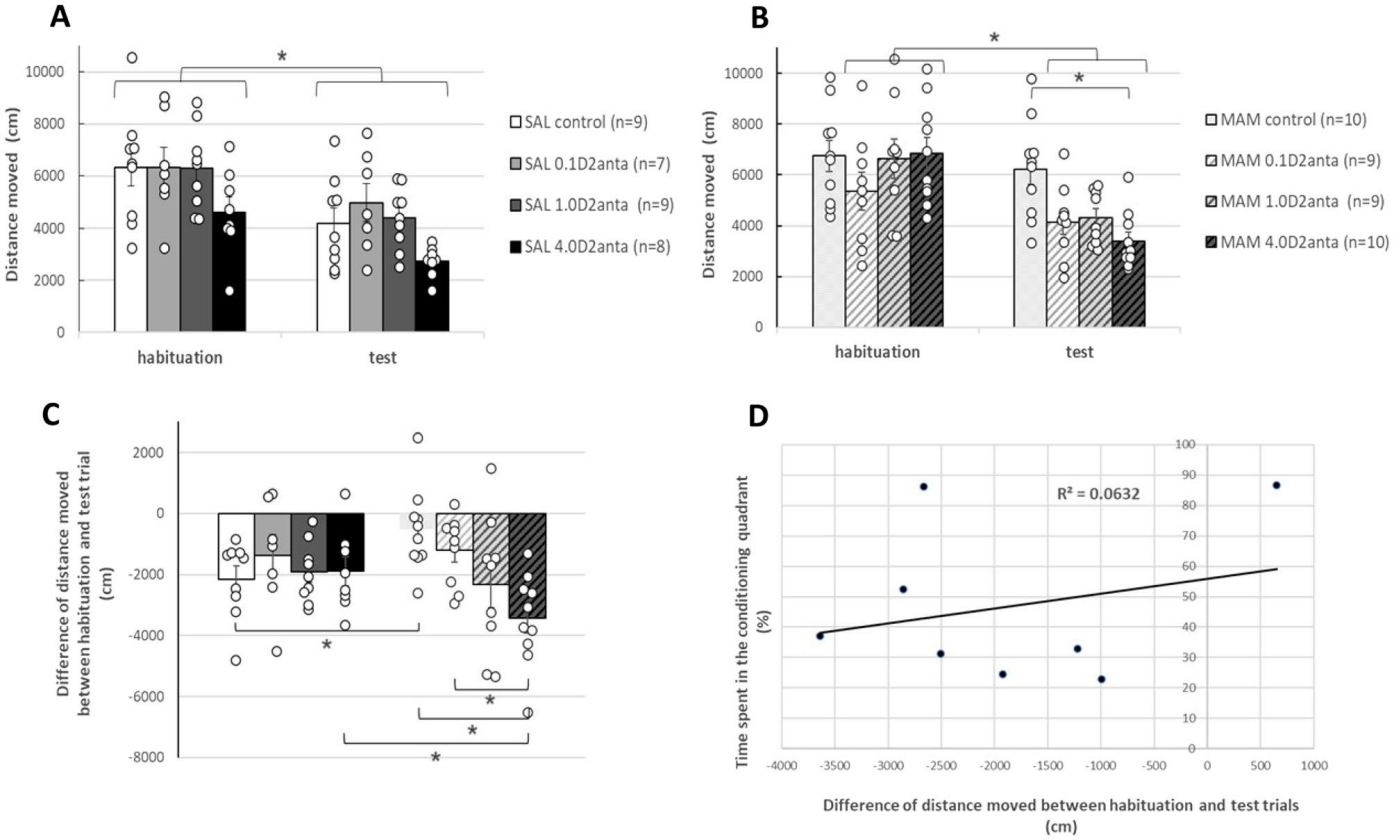
Effect of VP-sulpiride treatment on distance moved in SAL-E17 (**A**) and MAM-E17 rats (**B**) during habituation and test trials in the open field-based CPP paradigm. Panel (**C**) displays the difference in distance moved by the rats between the test and the habituation trials. The vehicle-treated MAM-E17 rats had a smaller (non-significant) decrease in locomotor activity from the habituation to the test trial compared to the vehicle-treated SAL-E17 rats. However, 4.0 µg dose of intra-VP sulpiride normalized the increased locomotor activity in MAM-E17 animals. Panel D shows that in the SAL-E17 4.0 µg sulpiride-treated group, there was no correlation between the distance moved and the time spent in the conditioning quadrant. The columns represent the average distance moved in cm (± S.E.M.) in panels (**A**) and (**B**), as well as the mean difference in distance moved in cm (± S.E.M.) between the test and habituation trials in panel (**C**). The results of the individual subjects (represented as circles) are added to the bar graphs. *p < 0.05 indicates significant differences.

In the same paradigm, the distance moved in the experimental arena by the rats was monitored, and the locomotor activity of the rats was also analyzed (see Fig. 5; for representative example tracks, see Fig. 3).

Three-way mixed ANOVA of the distance moved revealed a nonsignificant intrauterine MAM/vehicle treatment effect [F(1,71) = 1.748; p = 0.190], a significant drug treatment effect [F(3,71) = 3.132; p = 0.031] and a significant effect of trial [F(1,71) = 120.934; p = 0.001]. Intrauterine MAM/vehicle treatment x drug treatment interaction (F(3,71) = 2.256; p = 0.089), intrauterine MAM/vehicle treatment x trial interaction (F(1,71) = 0.015; p = 0.903) were not significant, while drug treatment x trial interaction (F(3,71) = 3.892; p = 0.012) was significant. Intrauterine MAM/vehicle treatment x drug treatment x trial interaction (F(3,71) = 4.027; p = 0.011) was significant as well. Post hoc test revealed that the MAM-E17 control rats moved significantly more in the test trial than the SAL-E17 control rats (p = 0.008). Furthermore, in the habituation trial there was a significant difference between the 4.0D2anta treated SAL-E17 and MAM-E17 rats (p = 0.005).

We have analyzed results of the SAL-E17 and MAM-E17 animals also separately, using Two-way mixed ANOVA.

Two-way mixed ANOVA of the distance moved by the SAL-E17 rats (Fig. 5A) revealed a significant drug treatment effect [F(3,33) = 3.054; p = 0.042], a significant effect of trial [F(1,33) = 68.239; p = 0.001], and a nonsignificant drug x trial interaction (F(3,33) = 0.524; p = 0.669). Post hoc tests revealed that SAL-E17 rats moved significantly less during the test trial than during the habituation trial (p = 0.001).

In the case of the MAM-E17 rats (Fig. 5B), however, two-way mixed ANOVA indicated a nonsignificant drug treatment effect [F(3,38) = 2.310; p = 0.092], a significant effect of trial [F(1,38) = 57.180; p = 0.001], and a significant drug x trial interaction (F(3,38) = 6.982; p = 0.001). Post hoc tests revealed that the locomotor activity of the MAM-E17 control animals was significantly greater than that of the 4.0 μg sulpiride-treated MAM-E17 group in the test trial (p = 0.003). Furthermore, locomotor activity in all groups, except for the control group, was significantly lower in the test trials than in the habituation trial (0.1 μg and 1.0 μg and 4.0 μg sulpiride-treated groups, p = 0.022, p = 0.001 and p = 0.001, respectively).

In the case of each animal, the difference in distance moved in the apparatus between the test and the habituation trials was also calculated and illustrated (Fig. 5C). Two-way ANOVA revealed a significant main effect of drug treatment [F(3, 63) = 3.453, p = 0.022], but there was no effect of intrauterine MAM/vehicle treatment [F(1, 63) = 0.013, p = 0.908]. However, there was a significant drug x intrauterine MAM/vehicle treatment interaction (F(3, 63) = 3.573, p = 0.019). The Bonferroni post hoc test revealed that in MAM-E17 rats, the data of 4.0 μg-treated rats were significantly different from those of vehicle-treated (p = 0.001) and 0.1 μg-treated rats (p = 0.011). Pairwise comparison revealed a difference between vehicle-treated SAL-E17 rats and vehicle-treated MAM-E17 rats (p = 0.021), which demonstrated that the decrease in locomotor activity in MAM-E17 rats without sulpiride treatment from habituation to the test was significantly lower than that in vehicle-treated SAL-E17 rats. In the case of the 4.0 μg sulpiride treatment group, there was also a difference between the SAL-E17 and MAM-E17 rats (p = 0.033).

Pearson’s test revealed that there was no correlation between the distance moved and the time spent in the conditioning quadrant in the SAL-E17 4.0D2anta group (R = 0.251; p = 0.548) (Fig. 5D).

## Discussion

In the present study, the behavioral effects of microinjection of the intra-VP D_2_R antagonist sulpiride were investigated in the open field-based CPP paradigm. Our results showed that the administration of sulpiride to the VP has a dose-dependent rewarding effect in SAL-E17 rats, which is consistent with our recent findings^29^. In contrast, in MAM-E17 rats, place preference could not be evoked by any dose of sulpiride. These results can be explained considering that the induction of place preference requires 1. the induction of a positive emotional state by the drug in the experimental animal, 2. the association of this positive “magnetic”/hedonic value with the environmental cues of the conditioning quadrant and 3. the consolidation of this associative memory in the central nervous system^35^. Spatial learning is an important component of learning strategies which can be used by rats in the CPP paradigm^36^. We have already demonstrated that intra-VP sulpiride can impair spatial learning processes in neurotypical rats^29,37^; nevertheless, this effect can be compensated by other non-spatial learning mechanisms, as we have shown previously^29^. However, because of the imbalanced subcortical DA metabolism in schizophrenia and schizophrenia model animals, we can suppose that compared with SAL-E17 rats, MAM-E17 rats are more sensitive to learning impairment caused by intra-VP sulpiride. This is supported by the fact that MAM-E17 animals exhibit learning/memory deficits in several paradigms^38,39^. What can be the mechanism by which the intra-VP sulpiride exerts its rewarding effect in the SAL-E17 rats? Stimulation of D_3_R-expressing VP neurons facilitates DAergic activity in the VTA, increasing DA levels in the NAC shell region and inducing place preference^40^. Based on these findings, it is plausible that the largest dose of sulpiride, eliminating the inhibitory effect of DA, can activate D_3_R-expressing VP neurons, facilitating VTA DAergic activity and consequently evoking place preference.

The main result of our present study concerns the locomotion-reducing effect of intra-VP sulpiride in MAM-E17 animals. It is well known that the increased locomotor activity of MAM-E17 rats can be paralleled with the positive symptoms of schizophrenia^9,10^. According to Kapur’s “salience” theory of positive symptoms^9^, in schizophrenia, the patient can assign aberrant salience and motivational significance to environmental events/cues^9^, leading to hyperreactivity and consequent psychomotor agitation. The increased locomotor activity of MAM-E17 rats might mimic the psychomotor agitation observed in schizophrenia^10^. The “dopamine hypothesis” of schizophrenia states that hyperresponsivity of the mesolimbic dopamine system underlies the pathophysiology of several symptoms, including positive symptoms^7^. D_2_R antagonists can effectively relieve the positive symptoms of this disease^41–43^; nevertheless, they can have biphasic effects over time. The acute systemic administration of D_2_R antagonists increases both the population activity and burst firing of DAergic neurons in (neurotypical) rats, while chronic administration of D_2_R antagonists results in a pronounced reduction in the number of spontaneously active DAergic neurons^17–20^. It was demonstrated that antipsychotics increase DAergic population activity via the NAC-VP–VTA feedback pathway, likely eliminating the inhibitory effect of DA on D_2_R-expressing NAC medium spiny neurons (MSNs), thus disinhibiting the VTA from VP inhibition^44^. Indeed, stimulation of D_2_R-expressing MSNs in the NAC increases VTA DAergic activity via the VP^23^. Nevertheless, based on these findings, we cannot explain how antipsychotics can evoke burst activity in DAergic neurons. It has been shown that phasic activation of VTA DAergic neurons induces place preference^45^. Phasic DAergic activation requires both an increased population and burst activity of DAergic neurons^24^; the former is regulated by the hippocampus-NAC-VP axis, while the latter is regulated by the pedunculopontine tegmental nucleus (PPTg)^46^. Accordingly, VP mainly regulates population activity^24^; however, we have shown recently that a high dose of the intra-VP D_2_R agonist quinpirole reduces both population and burst activity in the VTA^28^. It is reasonable to suppose that the intra-VP sulpiride can induce place preference, enhancing both population and burst activity, perhaps via D_3_R-expressing VP neurons (see above). In this way, the VP seems to be a plausible candidate where antipsychotics can exert their DAergic burst activity-enhancing effect. Interestingly, in contrast to the SAL-E17 rats, in the case of the MAM-E17 rats, the antipsychotic drugs immediately induced a depolarization block in the VTA DAergic neurons, reducing population activity^47^. This finding provides an alternative explanation for why conditioned place preference cannot be induced in MAM-E17 animals: there is no initial activation in the VTA population or burst activity. Nonetheless, the effect on locomotor activity is not acute, but it is lasting; it is present one day after the last sulpiride injection. What can be the underlying mechanism? The overactive hippocampus-NAC-VP-VTA pathway is responsible for the hyperresponsivity of the DAergic system^6,7^. D_2_R-expressing NAC-VP fibers facilitate VTA DAergic activity^23^, so we can suppose that this connection constitutes an essential element of the hippocampus-NAC-VP-VTA pathway. D_2_Rs can be found presynaptically on NAC fibers, terminating on VP neurons^25–27^. Therefore, we can hypothesize that sulpiride, which affects these presynaptic receptors, weakens the NAC-VP fibers, restoring population activity in the VTA and consequently reducing increased locomotion. This hypothesis is supported by the fact that systemic administration of D_2_R antagonists induces synaptic degeneration (which may be considered the endpoint of synaptic weakening) only in the VP, likely on fibers that project from the NAC to the VP^48^. In this way, in addition to the dopamine-cell depolarization block model^20^, we can identify a potential alternative mechanism by which D_2_R antagonist antipsychotic drugs can improve the positive symptoms of schizophrenia. The biphasic temporal regulation of VTA DAergic activity by VP D_2_Rs was also demonstrated by our recent finding, namely, that the activation of these receptors leads to initial inhibition but later (with a delay) to an increase in VTA population activity^28^.

It has been shown that the NAC-VP-VTA circuit plays an important role in the initiation of adaptive behavioral responses to novelty^49^, suggesting that these brain regions may be intimately involved in habituation processes. Furthermore, the VP is part of the hippocampus-NAC-VP-VTA loop that controls the entry of information into long-term memory^50^. In our recent paper, we suggested that intra-VP sulpiride can dose-dependently facilitate habituation processes^29^. In the present study, in the first so-called habituation trial, there was no difference between the SAL-E17 and MAM-E17 control groups. However, in the test trial, locomotor activity in the SAL-E17 control group was significantly lower than that in the MAM-E17 control group and was significantly lower than that in the first ‘habituation’ trial. The phenomenon observed in the case of the SAL-E17 rats is the well-known habituation process^51^. In contrast, in the case of MAM-E17 control rats, the distance moved decreased only minimally, reflecting habituation disturbance in these animals. However, the microinjection of larger doses of sulpiride into the VP restored habituation. Our present results suggest that in a novel environment, the increased locomotor activity of the MAM-E17 animals cannot be observed immediately but only after the habituation process. This is supported by several studies demonstrating that adult male MAM-E17 rats are not hyperactive when they are placed in a novel environment for the first time^3,21,52,53^. Furthermore, there are cases in which the same rats were investigated at different ages; thus, it cannot be decided whether the hyperactivity observed in late puberty and adulthood simply appears during these life periods or whether it is a habituation disturbance spanning ages^4,54,55^. Interestingly, when female rats were placed in a novel environment, they displayed hyperactivity^56^. A potential limitation of our present study is that only male but not female rats were investigated; further experiments are needed to elucidate the effects of intra-VP sulpiride on female rats.

The following question arises: Can habituation disturbance be regarded as a positive symptom? What is the difference between Kapur’s salience theory and the “habituation disturbance” theory? Initially, in a novel environment, many environmental cues can have transient but significant salience, which is suppressed by time; this process is called habituation. In this context, “habituation disturbance” can be interpreted as a special case of Kapur’s “salience” theory, in which the unreasonably increased and permanent salience of certain environmental cues can be due to insufficient habituational learning processes. A similar approach is suggested by Barkus et al. as well^57^. Consequently, habituation disturbance can result in positive symptoms. This is particularly important since disrupted habituation can be observed in the early stage of psychosis^58^, so it can be a prodromal symptom. This is also supported by the fact that patients with schizophrenia demonstrate habituation deficits^58–60^.

Overall, our present results are relevant for understanding the mechanisms underlying the symptoms of schizophrenia, thus facilitating the development of more effective therapies for this disease.

## Data Availability

The datasets generated during and/or analyzed during the current study are available from the corresponding author upon reasonable request.
